# Antiproliferative Activity of Crocin Involves Targeting of Microtubules in Breast Cancer Cells

**DOI:** 10.1038/srep44984

**Published:** 2017-03-24

**Authors:** Rupali R. Hire, Shalini Srivastava, Melissa B. Davis, Ananda Kumar Konreddy, Dulal Panda

**Affiliations:** 1 Department of Biosciences and Bioengineering, Indian Institute of Technology Bombay, Powai, Mumbai, 400076 India; 2 Department of Genetics, University of Georgia, Athens, Georgia 30602 USA; 3 Department of Biomedical & Pharmaceutical Sciences, University of Georgia College of Pharmacy, Athens, Georgia 30602 USA

## Abstract

Crocin, a component of saffron spice, is known to have an anticancer activity. However, the targets of crocin are not known. In this study, crocin was found to inhibit the proliferation of HCC70, HCC1806, HeLa and CCD1059sk cells by targeting microtubules. Crocin depolymerized both the interphase and mitotic microtubules of different cancer cells, inhibited mitosis and induced multipolar spindle formation in these cells. *In vitro*, crocin inhibited the assembly of pure tubulin as well as the assembly of microtubule-associated protein rich tubulin. Electron microscopic analysis showed that crocin inhibited microtubule assembly while it induced aggregation of tubulin at higher concentrations. Crocin co-eluted with tubulin suggesting that it binds to tubulin. Vinblastine inhibited the binding of crocin to tubulin while podophyllotoxin did not inhibit the crocin binding indicating that crocin binds at the vinblastine site on tubulin. The results suggested that crocin inhibited cell proliferation mainly by disrupting the microtubule network.

The digentiobiosyl ester of crocetin–α-crocin is a major component of saffron spice, derived from *Crocus sativus* L. It has been shown to have useful antioxidant^1^, anticonvulsant^2^, neuroprotective^3^, antiinflammatory^4^, antidepressant^5^ and antiproliferative^6^ properties. Crocin inhibits tumor growth and its spread in several forms of cancer including colorectal^7^, pancreatic^8^,^9^, breast^10^, and prostate cancers^11^, as well as chronic myelogenous^12^ and other leukemias^13^,^14^ indicating its potential use as a cancer chemotherapeutic and cancer preventive agent.

Crocin was found to inhibit cell cycle progression and to induce apoptosis^11^. However, the intracellular targets of crocin are not known. Crocin has been shown to induce apoptosis in tumor cells by down-regulating the expression of Bcl-2, survivin, cyclin D1 and up-regulating the expression of Bax in BALB/c xenograft tumor^15^. Distinct pro-apoptotic properties of crocin in human lung cancer were shown to act via the caspase-8-9-3 cascade^16^. A similar observation was made in apoptosis through the activation of caspases and enhancement of the Bax/Bcl-2 ratio in human gastric adenocarcinoma^17^. The strong cytotoxic effect on cultured human and animal adenocarcinoma cells exhibited a remarkable loss of cytoplasm and wide cytoplasmic vacuole-like areas^18^; cell shrinkage and pyknotic nuclei, suggesting apoptosis induction^19^.

Recently, it was indicated that crocin interacts with tubulin in a manner that increases the polymerization of microtubules *in vitro*^20^, however, the interaction within the cells has not yet been elucidated. Microtubules are found to be important targets of structurally different groups of antimitotic drugs that have been used with great success in the treatment of cancer. Tubulin binders either enhance or decrease the polymerization of microtubules and are known to perturb the dynamics of microtubules. Since microtubule dynamics play an important role in mitosis, this interference leads to blocking or inhibition of cell mitosis^21^. The best-known compounds that target microtubules are paclitaxel, colchicine and vinblastine.

In the present study, we have evaluated the anti-proliferative activity of crocin in cancer (HCC70, HCC1806 and HeLa) and normal (skin fibroblasts CCD1059sk) cells. Crocin inhibited the proliferation of cancer cells more efficiently than that of the non-cancer cells. It depolymerized both interphase and spindle microtubules and produced multipolar spindles in cancer cells. Crocin treatment was also found to block cell cycle progression at mitosis. Crocin bound to purified tubulin and inhibited the assembly of reconstituted microtubules *in vitro*. Podophyllotoxin did not inhibit the binding of crocin to tubulin. However, vinblastine inhibited the binding of crocin to tubulin and crocin increased the dissociation constant of the interaction between vinblastine and tubulin suggesting that crocin binds to the vinblastine binding site on tubulin. The evidence presented in the present work indicated that crocin inhibits cell proliferation by targeting tubulin. These results suggest that crocin has a potential to be developed as an anticancer agent.

## Results

### Effects of crocin on the proliferation of cancer cells

**Figure 1:**
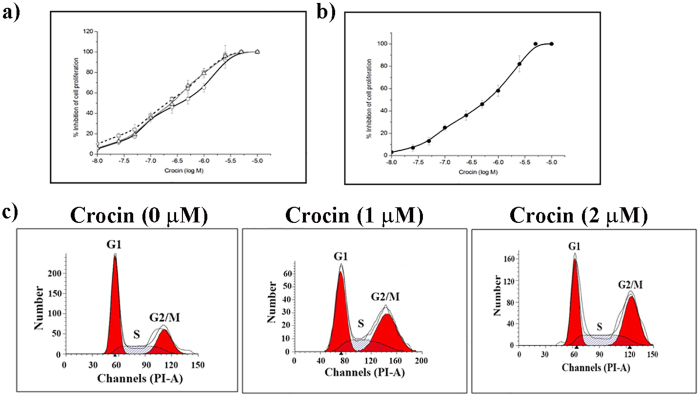
Crocin inhibited the proliferation of (**a**) cancer and (**b**) normal fibroblasts cells. Cancer cell types HeLa (○), HCC1806 (□), HCC70 (Δ) and normal fibroblasts CCD1059sk (●) were incubated with different concentrations of crocin for 24 h. The cell proliferation was determined by counting dead and live cells with trypan blue. Three sets of experiments were performed and the error bars indicate the standard deviation. (**c**) The cell cycle analysis of HeLa cells treated without and with 1 and 2 μM crocin for 24 h. DNA was stained with PI and analyzed by FACS. The experiment was performed twice.

**Figure 2:**
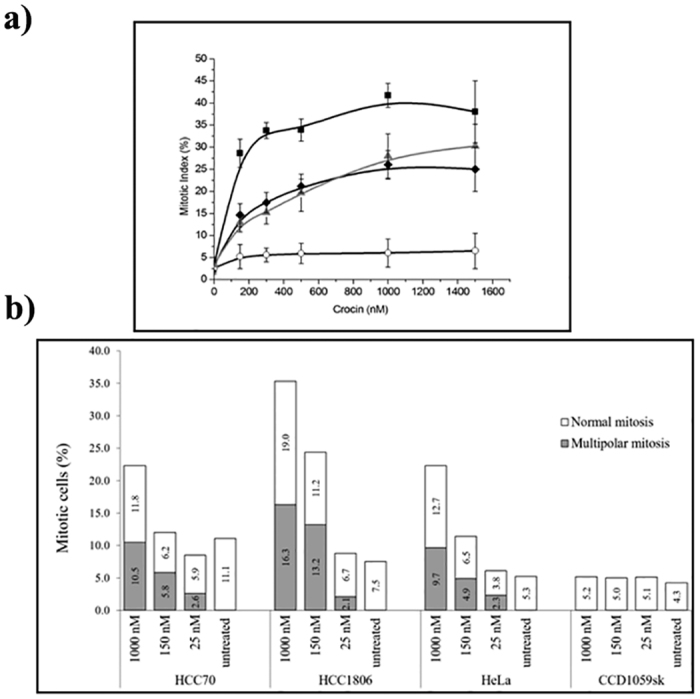
Crocin induced mitotic block and produced multipolar spindles in cancer cells. (**a**) HCC1806 (■), HCC70 (♦) HeLa (Δ) and normal fibroblast CCD1059sk (○) were treated with increasing concentration of crocin and the percentage of cells in mitosis were determined. In each case 300 cells were counted. Error bars indicate the SD. (**b**) Crocin increased the number of multipolar mitotic cells in cancer cell lines. The percentage of normal (grey bars) and multipolar (black bars) mitotic cells are shown when treated with different concentrations of crocin.

Crocin inhibited the proliferation of HCC70, HCC1806, HeLa, and CCD1059sk cells in culture (Fig. 1a and 1b). The half-maximal proliferation inhibitory concentration (IC_50_) of crocin was determined to be 209 ± 5, 275 ± 8, 380 ± 7 and 660 ± 23 nM for HCC1806, HCC70, HeLa, and CCD1059sk cells, respectively (Fig. 1a and b). The effect of crocin on the cell cycle of HeLa cells was analyzed using FACS (Fig. 1c). The percentage of cells in the G2/M phase was determined to be 26, 52 and 58% in the absence and presence of 1 and 2 μM crocin, respectively. Since crocin inhibited HeLa cell cycle progression in the G2/M phase, the effect of crocin on the mitotic index (% of cell in mitosis) of HCC1806, HCC70, HeLa, and CCD1059sk cells was examined (Fig. 2a). Crocin treatment increased the mitotic index in HCC1806, HCC70, and HeLa cells. However, there was no significant increase in the mitotic index of CCD1059sk cells. For example, the mitotic index in HCC1806 cells increased from 3.2 ± 1.1% to 41.7 ± 2.7% after exposure to 1 μM crocin for 24 h while the mitotic index in CCD1059sk cells increased from 2.5 ± 1.3% to 6.0 ± 4% under similar conditions. The mitotic spindles in the crocin-treated cells were carefully examined (Fig. 2b). Many multipolar spindles were formed in the crocin-treated HCC1806 cells. For example, 45% of the mitotic population was multipolar in the presence of 1000 nM crocin. Similarly, a significant increase in the multipolar spindles was observed in HeLa and HCC70 cells after crocin treatment. However, the number of multipolar spindles did not increase significantly in crocin-treated normal CCD1059sk cells.

### Crocin perturbed the organization of microtubules in the interphase cells

**Figure 3:**
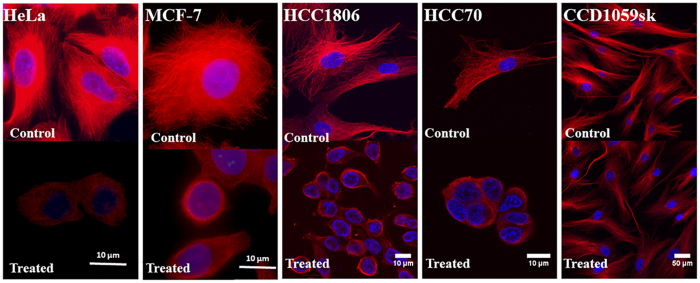
Effects of crocin on the interphase microtubules. The cancer cells and normal fibroblast cells were incubated in the absence (control) or the presence (treated) of 1 μM crocin for 24 h. Microtubules are shown in red and the nuclear stain is shown in blue.

Crocin depolymerized the interphase microtubule network in different cancer cells (Fig. 3). The immunostaining of HeLa cells with α-tubulin IgG showed regular microtubule network. The microtubules were well spread with distinct filaments. Crocin (1000 nM) treated HeLa cells showed significant microtubule depolymerization with diffuse staining of α-tubulin. Similar effects were observed in MCF-7, HCC70 and HCC1806 cells. However, no significant microtubule depolymerizing effect of crocin was observed in CCD1059sk cells.

### Effect of crocin on the mitotic cells

**Figure 4:**
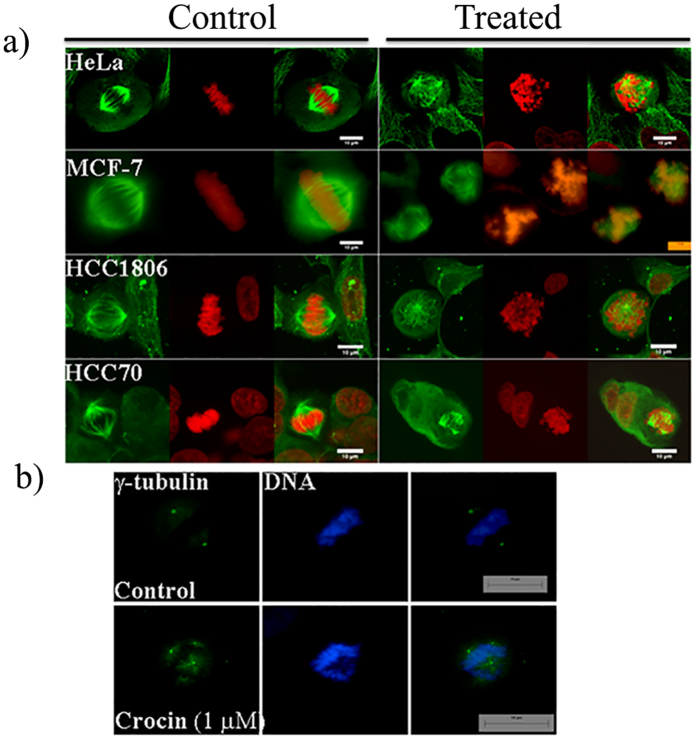
Crocin treatment induced multipolar spindle formation and caused chromosomal misalignment in the mitotic cells. (**a**) Mitotic spindles of HeLa, MCF-7, HCC70, HCC1806 cells in the absence (control) and presence of 1.0 μM crocin for 24 h are shown. Microtubules are shown in green and DAPI stained nuclei or chromosomes are shown in red. (**b**) HeLa cells were treated without and with crocin (1 μM) for 24 h and immunostained with γ-tubulin IgG and Hoechst (DNA). The scale bar indicates a length of 10 μm.

Because crocin treatment induced a mitotic block in cancer cells, the effects of crocin on the mitotic spindles morphology were analyzed. Crocin treatment depolymerized spindle microtubules in HeLa, MCF-7, HCC70 and HCC1806 cells and induced the formation of multipolar spindles in these cells (Fig. 4a, Treated). The cancer cells displayed multi-astral disrupted spindles and the number of astral bodies was two or more at the spindle poles upon crocin exposure. These spindle defects caused the chromosomes to missegregate and misalign, preventing the formation of organized equatorial metaphase plate that is observed in normal mitosis (Fig. 4a, Control). The chromosomes were either spread out in some cells where bipolar spindle was observed or pulled at the poles closer to the centrosome in the multipolar cells leaving some of the chromosomes at the cell equator. The results indicated that crocin disrupted the mitotic spindles and caused chromosome misalignment. Further, the effect of crocin treatment on centrosomes in HeLa cells was examined (Fig. 4b). Two γ-tubulin foci at two opposite poles of the cell were observed in the control cells. However, centrosomes were found to be fragmented and multiple γ-tubulin foci were observed in the crocin-treated HeLa cells suggesting the de-clustering of the centrosomes (Fig. 4b).

### Crocin inhibited microtubule assembly *in vitro*

**Figure 5:**
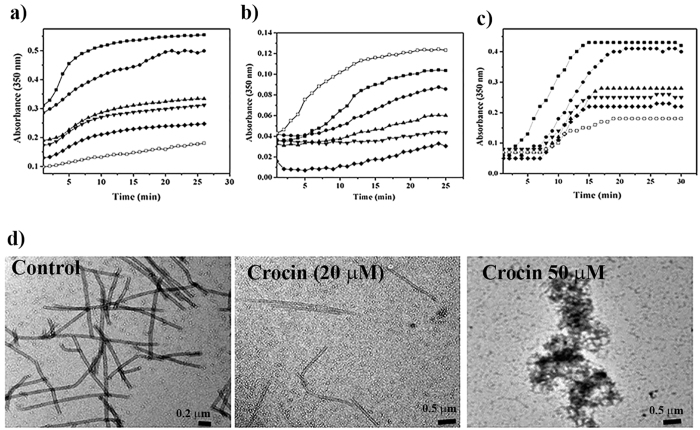
Crocin inhibited the assembly of tubulin *in vitro*. Tubulin (10 μM) was incubated in the absence (■) and presence of different concentrations (10 (●), 20 (▲), 30 (▼), 40 (♦) and 50 (□) μM of crocin for 5 min on ice in PEM buffer containing either 1 M monosodium glutamate (**a**) or 10% DMSO (**b**). Further, GTP (1 mM) was added to the reaction mixtures. The assembly of microtubules was monitored by light scattering at 350 nm. (**c**) MAP-rich (2 mg/mL) tubulin was incubated in the absence (■) and presence of different concentrations (10 (●), 20 (▲), 30 (▼), 40 (♦) and 50 (□) μM) of crocin for 10 min on ice. Subsequently, 1 mM GTP was added to the reaction mixtures and the assembly of MAP-rich tubulin was monitored at 37 °C. The experiment was performed three times. (**d**) Tubulin (10 μM) was polymerized in the absence and presence of 20 and 50 μM crocin for 10 min with 1 M monosodium glutamate and 1 mM GTP. Electron micrographs of polymers in the absence and presence of crocin are shown. The scale bars are shown in the figures.

The effect of crocin on the kinetics of purified tubulin polymerization was monitored by light scattering. Crocin inhibited the initial growth rate of glutamate-induced tubulin polymerization indicating that it suppressed the nucleation of microtubules (Fig. 5a). Further, crocin inhibited the assembly of tubulin in a concentration dependent manner (Fig. 5a). The half-maximal inhibitory concentration of tubulin polymerization by crocin was determined to be 38 ± 6 μM. Similarly, crocin also inhibited tubulin assembly in the presence of DMSO (Fig. 5b). In the presence of DMSO, crocin inhibited tubulin assembly at a lower concentration; while tubulin aggregation was induced at higher concentration of the compound. Further, crocin also inhibited the assembly of microtubule associated protein (MAP) rich tubulin in a concentration dependent manner (Fig. 5c). The lag phase of MAP-rich tubulin assembly increased significantly in the presence of crocin indicating that it hindered the nucleation step of the assembly of microtubules. The assembly of MAP-rich microtubules was inhibited by 50% in the presence of 42 ± 5 μM crocin. In addition, the electron micrographs also showed that crocin inhibited glutamate-induced tubulin assembly (Fig. 5d). In the presence of 50 μM crocin, tubulin-aggregates were found suggesting that crocin at lower concentration inhibited the polymerization of tubulin and at higher concentration induced tubulin aggregation (Fig. 5d).

### Crocin binds to purified tubulin *in vitro*

**Figure 6:**
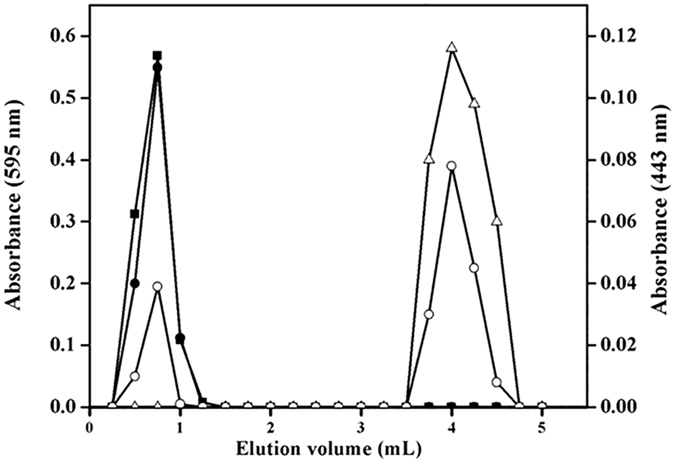
Crocin bound to purified tubulin *in vitro*. The elution profiles of free 20 μM tubulin (■) and 120 μM crocin (Δ) are shown. Tubulin (20 μM) was incubated with 120 μM crocin for 30 min at 25 °C and then, eluted through the same column. The elution of tubulin (●) and crocin (○) are shown from combination of tubulin-crocin. The experiment was performed three times.

The interaction between crocin and tubulin was examined by gel filtration chromatography. Tubulin and crocin were eluted at 0.5 and 4.25 mL, respectively, when loaded separately (Fig. 6). In the mixture of tubulin and crocin, tubulin was eluted as a single peak at 0.5 mL and crocin was eluted with two different elution volumes; one peak was at ~0.5 mL and the another peak was at 4.25 mL. The elution profile suggested that crocin co-eluted with tubulin and indicated that crocin binds to tubulin *in vitro*. Further, the stoichiometry for the binding of crocin to tubulin was determined to be 1.1 ± 0.4.

### Crocin binds at the vinblastine site in tubulin

**Figure 7:**
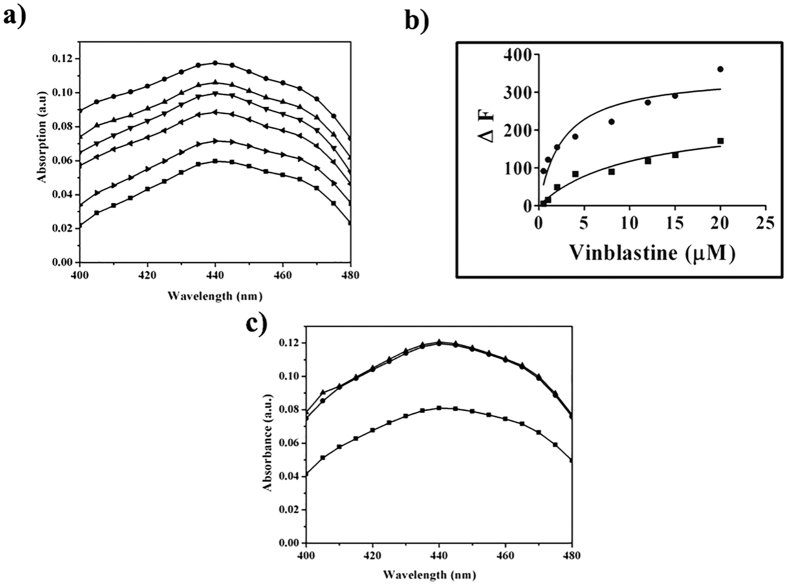
Effects of vinblastine and podophyllotoxin on the binding of crocin to tubulin. (**a**) Tubulin (10 μM) was first incubated without or with different concentrations of vinblastine and then, 10 μM crocin was added to the reaction mixtures. The absorption spectra of crocin (10 μM) in the absence (■) and presence of tubulin (●) are shown. The absorption spectra of tubulin-crocin complex in the presence of increasing concentrations of vinblastine 3 (▲), 5 (▼), 10 (◄) and 15 (►) μM are also shown. The experiment was done 3 times. (**b**) The change in the tryptophan fluorescence intensity was plotted against different concentrations of vinblastine in the absence (●) and presence of 25 μM crocin (■). The data were fitted in a binding equation as mentioned in the method section. The experiment was done 4 times. (**c**) Tubulin (10 μM) was first incubated without or with 15 μM podophyllotoxin and then, 10 μM crocin was added to the reaction mixtures. The absorption spectra of free crocin (10 μM) (■), crocin-tubulin (●) complex without podophyllotoxin and crocin-tubulin complex (▲) in the presence of 15 μM podophyllotoxin are shown. The experiment was performed 3 times.

Microtubule depolymerizing agents are reported to bind to tubulin either at the colchicine or the vinblastine site on tubulin^21^. Therefore, we examined whether crocin could bind to either of these sites. The absorption spectra of crocin showed its maxima at 443 nm. In the presence of tubulin, the absorption of crocin was found to increase indicating that crocin binds to tubulin (Fig. 7a). We checked whether vinblastine could inhibit the binding of crocin to tubulin (Fig. 7a). The pre-incubation of vinblastine with tubulin inhibited the binding of crocin to tubulin indicating that crocin binds to the vinblastine site on tubulin (Fig. 7a). Further, we determined the binding affinity of vinblastine to tubulin in the absence and presence of 25 μM crocin using intrinsic tryptophan fluorescence of tubulin (Fig. 7b). The dissociation constant (K_d_) for the binding of vinblastine to tubulin was determined to be 2.5 ± 0.5 and be 9.5 ± 5.3 μM in the absence and presence of 25 μM crocin, respectively. The increase in the dissociation constant of vinblastine-tubulin interaction by ~3.8 times in the presence of crocin further indicated that crocin binds to tubulin at the vinblastine site. Podophyllotoxin, a well-known colchicine site agent^22^, did not inhibit the increase of the absorption of crocin in the presence of tubulin indicating that podophyllotoxin does not inhibit the binding of crocin to tubulin (Fig. 7c).

## Discussion

In this study, crocin, a major carotenoid component of the saffron spice, was found to target tubulin/microtubules and to inhibit the proliferation of different types of cancer cells in culture. Macular carotenoids such as lutein and zeaxanthin are reported to bind to retinal tubulin^23^. Our *in vitro* studies showed that, upon interaction with tubulin, crocin inhibited the assembly of microtubules. The treatment of cells with crocin led to the formation of multipolar spindles with distorted chromosomes and centrosome fragmentation similar to the cells treated with well-known anti-mitotic drugs such as vinblastine, paclitaxel and cryptophycin 52^21^. The cancer cells were significantly more susceptible to crocin treatment than the non-cancerous fibroblast cells. The results indicated that the anti-proliferative action of crocin involves perturbation of mitosis in cancer cells. The presence of multi polar cells at a concentration of 150 nM which is below the IC_50_ values of the cancer cells, suggests that some of the multipolar cells may exit mitosis without undergoing apoptosis. Further, the crocin-treated interphase cells displayed a dense perinuclear microtubule network similar to those seen in the presence of vincristine, a microtubule-depolymerizing agent^24^. This also suggests that the microtubules are targeted, an effect that has been observed in cases where HeLa cells were exposed to crocin in early studies^19^. Crocin treated cancer cells such as HeLa, MCF-7, HCC70 and HCC1806 displayed a higher number of multipolar mitotic cells than that of noncancerous fibroblast cells. In addition, the microtubules of cancer cells were significantly perturbed upon crocin treatment while the microtubules of normal fibroblast cells were relatively unaffected under the similar treatment conditions. This observation needs to be further investigated to address the question whether crocin specifically inhibits cells undergoing rapid mitosis such as cancer cells.

### Crocin inhibits tubulin assembly

Crocin was shown to inhibit the proliferation of a variety of cancer cells^8^,^25^,^26^. It was indicated that crocin enhances the assembly of microtubules from the increase in the light scattering signal of tubulin assembly in the presence of crocin^20^. However, the increase in the light scattering in the presence of high concentration of crocin was likely to be due to the aggregation of tubulin because a concentration of tubulin lower than the critical concentration of tubulin polymerization was used in the study^20^. In the present work, crocin was found to inhibit tubulin polymerization; the compound inhibited the assembly of purified tubulin in the presence of either DMSO or glutamate. Further, it also inhibited the assembly of MAP-rich tubulin. Though crocin inhibited microtubule assembly at low concentrations, crocin was found to induce the aggregation of tubulin *in vitro* and in cultured cells at relatively high concentrations. Several microtubule targeting compounds such as cryptophycin, dolastatin, vinblastine and griseofulvin inhibit tubulin polymerization at low concentrations and induce aggregation of tubulin at high concentration^27^,^28^.

### Mechanism of inhibitory action of crocin

**Figure 8:**
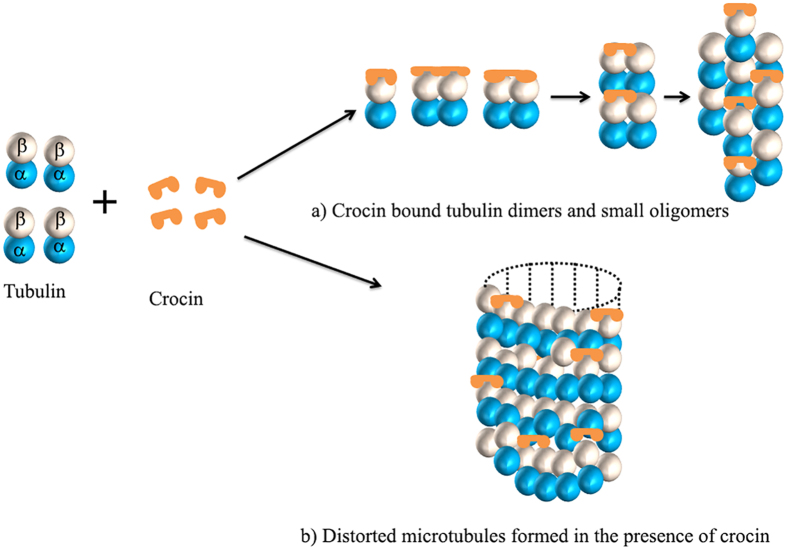
A model showing the effect of crocin on tubulin polymerization. Crocin can bind to a tubulin dimer. Alternatively, a long and flexible crocin molecule may bind to two tubulin dimers as shown. Crocin can inhibit microtubule polymerization either (**a**) by inducing the formation of tubulin oligomers and aggregates or (**b**) by producing defective microtubules. Crocin can inhibit the addition of tubulin dimers at the growing end of a microtubule by causing steric hindrance. In addition, the presence of several crocin molecules into a microtubule could produce significant distortion in the microtubule lattice leading to its disassembly.

Similar to vinblastine, crocin inhibited tubulin polymerization at low concentration but produced tubulin aggregates at relatively high concentrations. The binding experiments suggested that crocin competes with vinblastine for its binding to tubulin. We propose that the mode of action of crocin is similar to that of vinblastine (Fig. 8). Vinblastine binds reversibly to the β-subunit of tubulin dimers at a site called as *Vinca* binding site^28^,^29^,^30^. The crystal structure of vinblastine bound to tubulin showed that vinblastine binding to tubulin forms a wedge at the interface of two tubulin molecules and inhibits the assembly of microtubules^30^. The binding of vinblastine to tubulin induces a conformational change in tubulin, which increases the affinity of tubulin for itself; thereby, promoting self-association or aggregation of tubulin^31^,^32^,^33^,^34^. Vinblastine also binds to the exposed tubulin in the microtubules with a higher affinity than the one which is buried deep inside the microtubule lattice^28^,^35^,^36^. Crocin, like vinblastine, can induce conformational change in tubulin. The perturbed tubulin dimers could oligomerize to form aggregates (Fig. 8a). Crocin is a long and flexible molecule, and thus, one molecule of crocin can bind to two tubulin dimers and thereby, it can induce self-association of tubulin. Like vinblastine^36^, crocin could also bind to the end of microtubules. The binding of crocin to the ends of microtubules can perturb the end structures of the microtubules (Fig. 8b). Further, the binding of several crocin molecules to the microtubule lattice can distort microtubules and eventually lead to the disassembly of microtubules.

### Crocin as an anticancer therapeutic drug

Several microtubule-targeting highly successful anticancer drugs such as vinblastine, vincristine and paclitaxel are natural products^28^. Crocin is also a natural product and being a constituent of edible spice saffron, it is also nontoxic at low concentration for human consumption. Crocin exhibits harmful effects in animal above a dose of 100 mg/kg of body weight^16^. The saffron extract dosage upto 5 g/Kg of body weight was found to be non-toxic in mice^6^. With many known medicinal benefits due to its anti-oxidant, anti-depressant, and anticonvulsant properties, it is useful for a wide spectrum of diseases. Interestingly, crocin seems to be more active against cancer cells than the non-cancerous cells indicating that crocin has a potential to be a successful anticancer agent.

## Methods

### Materials

1,4-piperazinediethanesulfonic acid (PIPES); 2-(N-morpholino) ethanesulfonic acid (MES); ethylene glycol-bis (2-aminoethylether)-*N,N,N′,N′*-tetraacetic acid (EGTA); 4’,6-diamidino-2-phenylindole (DAPI); propidium iodide (PI); GTP and crocin were obtained from Sigma (St. Louis, MO USA). All other reagents were of analytical grade.

### Tubulin Purification

Microtubules were prepared from goat brains using PEM (50 mM PIPES, 1 mM EGTA, 2 mM MgSO4, pH 6.9) as the homogenization buffer^37^. Following the homogenization, microtubules were extracted using two cycles of polymerization and depolymerization in the presence of 1 M monosodium glutamate and 10% (v/v) dimethyl sulfoxide (DMSO)^38^. For the purification of MAP-rich tubulin, two cycles of polymerization were performed using 4 M glycerol^39^. The proteins were then stored at −80 °C. The Bradford reagent (Bio-Rad, Hercules, USA) was used to determine the protein concentration using BSA as a standard^40^.

### Tubulin Polymerization Assay

Goat brain tubulin (10 μM) was incubated without and with different concentrations (0–50 μM) of crocin in an assembly buffer (25 mM PIPES at pH 6.8, 3 mM MgCl_2_, 1 mM EGTA) with 1 M monosodium glutamate on the ice. Subsequently, 1 mM GTP was added to the reaction mixtures and the assembly kinetics was monitored at 37 °C by light scattering at 350 nm using a Multi plate reader Spectra Max. The effect of crocin on the DMSO-induced assembly of tubulin was examined similarly using 10% DMSO instead of 1 M monosodium glutamate. Further, the effect of crocin on the assembly of MAP-rich tubulin was examined. MAP-rich tubulin (2 mg/mL) was polymerized in the presence of 1 mM GTP in 25 mM PIPES at pH 6.8, 3 mM MgCl_2_, 1 mM EGTA without or with different concentrations of crocin for 30 min. The experiment was performed three times in each case.

### Transmission Electron Microscopy

Tubulin (10 μM) was incubated without and with crocin (20 and 50 μM) for 10 min in PEM buffer (50 mM PIPES, 3 mM MgCl_2_, 1 mM EGTA) with 1 M monosodium glutamate on ice. Subsequently, 1 mM GTP was added and polymerized for 10 min at 37 °C. The samples were then spotted on formvar carbon coated copper grids (300 mesh) for 40 sec. The grids were then washed with MilliQ water and dried for 10 min. Further, uranyl acetate (1%) was applied on to the grids for 40 sec followed by washing with MiliQ water. The grids were then observed under a JEM 2100 Ultra HRTEM instrument at 200 kV.

### Cell Culture and Proliferation Assay

Cultures of HeLa (cervical cancer), HCC70, MCF-7, HCC1806 (various types of breast cancer) and CCD1059sk (normal fibroblasts cells) were used to test the effect of crocin on cell proliferation. The cancer cell lines were grown and maintained in Minimal Essential Medium (ATCC) supplemented with 10% (v/v) fetal bovine serum and the fibroblasts were grown in Eagle’s minimal essential medium (ATCC) supplemented with 10% fetal bovine serum. The stock crocin was prepared in 1% methanol in PBS. The cultures were grown at 37 °C in 5% CO_2_. Cell proliferation was determined by counting cells. Briefly, 5000 cells/well were seeded and incubated without and with increasing concentrations of crocin. Both trypsin treated adherent and floating cells were combined and counted after the addition of 0.4% final concentration of trypan blue. A minimum of 36 WBC grids in a hemocytometer was counted for each reaction condition and each sample was counted in duplicates. Growth inhibition (GI) was calculated as a percentage of untreated controls. The inhibition was plotted against the log of crocin concentration and the IC_50_ values were interpolated from the resulting linear regression curve fit. Experiments were performed three times in duplicates for each cell line.

### Cell Cycle Analysis

HeLa cells were incubated without and with crocin (1 and 2 μM) for 24 h. Further, the cells were rinsed thoroughly twice with PBS. The cells were permeabilized with chilled 70% ethanol for 30 min at 4 °C. Washing was done with chilled PBS twice and cells were incubated with 30 μg/mL of PI and RnaseA (10 μg/mL) for 2 h on ice^41^. The analysis was done using BD FACS Aria system (Becton Dickinson, San Jose, CA, USA).

### Mitotic Index

Cells (1 × 10^5^ cells per well) were grown on poly-l-lysine coated cover slips in 6-well tissue culture plates. The cells were incubated with different concentrations ranging from 0–1500 nM of crocin in media for 24 h. Both floating and adherent cells were collected on coverslips by centrifugation at 1000 × g and then fixed with 2% formaldehyde. The mitotic index was determined by staining the cells with DAPI (1 μg/mL). The cells were counted using an EVOS fluorescence microscope (Advanced microscopy group, USA) with a 40X objective. A minimum of 300 cells was counted for each concentration of crocin per experiment. Multipolar cells were counted in a separate experiment after exposing the pre-adhered cells of both cancer and normal fibroblasts to 25, 150 and 1000 nM of crocin for 16 h. The multipolar cells were observed with EVOS fluorescence microscope at 40X after staining with anti-tubulin antibodies and DAPI.

### Immunofluorescence Microscopy

Immunofluorescence microscopy was performed as described previously^42^. Briefly, the cells were grown on coverslips to a density of 1 × 10^5^ cells/mL and treated with 1 μM crocin for 24 h. Cells were fixed in 3.7% formaldehyde for 30 min at 37 °C, and then transferred to cold (−20 °C) methanol for 10 min. Blocking was done using 2% BSA in PBS for 15 min and the mouse monoclonal anti- α tubulin IgG antibody diluted (1:150) or anti-mouse γ-tubulin IgG (1:500) in BSA/PBS was added for 2 h. Washing (3 times) was done by BSA/PBS followed by the addition of Alexa-568-labeled anti-mouse IgG (1:100) for 1 h. Coverslips were rinsed with 2% BSA/PBS for 10 min and mounted using a mounting media containing DAPI (Electron microscopy, USA). The microtubules and chromosomes were observed with a Zeiss LSM 710 confocal system with 100X objective and a Nikon Eclipse TE2000-U microscope. The images were analyzed using Image-Pro Plus software (Media Cybernetics, MD, USA) for IF images. The IF images obtained from the confocal microscope were processed and analysed using ZEN lite software (Carl Zeiss USA). The numbers of cells undergoing mitosis following treatment with different concentrations of crocin were counted using the EVOS florescent microscope and ImageJ software by scoring at least 200 cells per well or 20 fields.

### Gel Filtration Chromatography

Tubulin (20 μM) was incubated with crocin (120 μM) for 30 min at 25 °C in 25 mM PIPES (pH 6.8). Tubulin, crocin and tubulin- crocin complex were loaded individually onto the P4 resin and fractions of 250 μL were collected. Tubulin was monitored using Bradford reagent by measuring absorbance at 595 nm. Crocin was detected by monitoring the absorbance at 443 nm. The stoichiometry of crocin binding to tubulin was estimated from the concentration of tubulin and crocin in the mixture. The molar extinction coefficient of crocin at 443 nm was determined to be 5000 M^−1^ cm^−1^. The experiment was performed 3 times.

### Determination of crocin binding site

Crocin (10 μM) was incubated in the absence or presence of 10 μM tubulin in 25 mM PIPES buffer, pH 6.8. The absorption spectra (400 to 480 nm) were recorded using a spectrophotometer (Jasco, V-730). To check whether crocin binds to tubulin at the colchicine binding site, tubulin was first incubated with 15 μM podophyllotoxin for 5 min at 25 °C. Then, the mixture was further incubated with10 μM crocin for 10 min and the absorption spectra were monitored. In a separate experiment, tubulin was first incubated without or with several concentrations of vinblastine (3, 5, 10 and 15 μM) for 5 min at 25 °C. Then, the reaction mixtures were incubated with 10 μM crocin for 10 min at 25 °C. The absorption spectra of tubulin in the presence of increasing concentrations of vinblastine without crocin were also measured. The experiment was repeated 3 times in each case.

### Effects of crocin on the binding of vinblastine to tubulin

Tubulin (2 μM) was incubated without or with different concentrations (0.5 to 20 μM) of vinblastine in 25 mM PIPES buffer for 30 min at 30 °C. The tryptophan fluorescence spectra (310–370 nm) were recorded by exciting the mixtures at 295 nm. The fluorescence intensities were corrected for inner filter effect as described previously^43^ and the corrected fluorescence intensities were used to determine the fluorescence changes (∆F) in the presence of different concentrations of vinblastine. The dissociation constant of the binding of vinblastine to tubulin was determined using an equation,


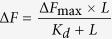


Where, ΔF is the change in the tryptophan fluorescence intensity in the presence of vinblastine, ΔF_max_ is the maximal change in the fluorescence intensity, and L is the concentration of vinblastine. The data were fitted using Graph Pad Prism 6 software^44^. In a separate experiment, tubulin (2 μM) was incubated with crocin (25 μM) for 10 min in 25 mM PIPES buffer. Then, the reaction mixtures were incubated without or with different concentrations (0.5 to 20 μM) of vinblastine for 30 min at 30 °C. The fluorescence spectra were recorded and the dissociation constant was calculated as described above. The experiment was done four times in each case.

## Additional Information

**How to cite this article**: Hire, R. R. *et al*. Antiproliferative Activity of Crocin Involves Targeting of Microtubules in Breast Cancer Cells. *Sci. Rep.* **7**, 44984; doi: 10.1038/srep44984 (2017).

**Publisher's note:** Springer Nature remains neutral with regard to jurisdictional claims in published maps and institutional affiliations.
